# Activators and Inhibitors of Protein Kinase C (PKC): Their Applications in Clinical Trials

**DOI:** 10.3390/pharmaceutics13111748

**Published:** 2021-10-20

**Authors:** Takahito Kawano, Junichi Inokuchi, Masatoshi Eto, Masaharu Murata, Jeong-Hun Kang

**Affiliations:** 1 Center for Advanced Medical Innovation, Kyushu University, 3-1-1 Maidashi, Higashi-ku, Fukuoka 812-8582, Japan; t-kawano@dem.med.kyushu-u.ac.jp (T.K.); etom@uro.med.kyushu-u.ac.jp (M.E.); 2 Department of Urology, Graduate School of Medical Sciences, Kyushu University, 3-1-1 Maidashi, Higashi-ku, Fukuoka 812-8582, Japan; junichi@uro.med.kyushu-u.ac.jp; 3 Division of Biopharmaceutics and Pharmacokinetics, National Cerebral and Cardiovascular Center Research Institute, 6-1 Shinmachi, Kishibe, Suita 564-8565, Japan

**Keywords:** protein kinase C, cancer, inhibitor, activator, signaling pathway, clinical trial

## Abstract

Protein kinase C (PKC), a family of phospholipid-dependent serine/threonine kinase, is classed into three subfamilies based on their structural and activation characteristics: conventional or classic PKC isozymes (cPKCs; α, βI, βII, and γ), novel or non-classic PKC isozymes (nPKCs; δ, ε, η, and θ), and atypical PKC isozymes (aPKCs; ζ, ι, and λ). PKC inhibitors and activators are used to understand PKC-mediated intracellular signaling pathways and for the diagnosis and treatment of various PKC-associated diseases, such as cancers, neurological diseases, cardiovascular diseases, and infections. Many clinical trials of PKC inhibitors in cancers showed no significant clinical benefits, meaning that there is a limitation to design a cancer therapeutic strategy targeting PKC alone. This review will focus on the activators and inhibitors of PKC and their applications in clinical trials.

## 1. Introduction

**Figure 1:**
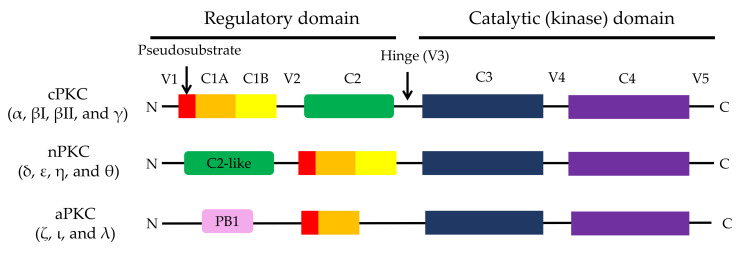
Structure of PKC isozymes. All PKCs consist of a regulatory domain, a catalytic (kinase) domain, and variable regions (V1–V5). The regulatory domain of all PKCs includes a C1 domain with a pseudosubstrate motif. Additionally, cPKCs, nPKC, and aPKCs have a C2 domain that binds to Ca^2+^, a C2-like domain that cannot bind to Ca^2+^, and a PB1 domain in the regulatory region, respectively. The C3 and the C4 domain of all PKCs bind to ATP and substrate, respectively. Reproduced with permission from Kang, J.H. et al., Biotechnol. Adv.; published by Elsevier, 2012.

Protein kinase C (PKC) is a family of phospholipid-dependent serine/threonine kinases and is classified into three subfamilies based on their structural and activation characteristics: conventional or classic PKC isozymes (cPKCs; α, βI, βII, and γ), novel or non-classic PKC isozymes (nPKCs; δ, ε, η, and θ), and atypical PKC isozymes (aPKCs; ζ, ι, and λ) (Figure 1) [1,2,3]. Consensus phosphorylation site motifs for PKCs are (R/K)X(S/T), (R/K)(R/K)X(S/T), (R/K)XX(S/T), (R/K)X(S/T)XR/K, and (R/K)XX(S/T)XR/K, meaning that their substrates are typically rich in basic amino acids (arginine (R) and/or lysine (K)) [4].

PKCs are involved in multiple signal transduction systems that control cell proliferation, differentiation, survival, invasion, migration, and apoptosis. For these reasons, PKCs are regarded as important targets for the treatment of various diseases, such as cancers, neurological diseases (e.g., Alzheimer’s disease (AD)), cardiovascular diseases (e.g., heart failure), and infections (e.g., acquired immunodeficiency syndrome) (for review see [2,5,6,7]). PKC inhibitors and activators can be used for the treatment of various PKC-associated diseases. In this review, we will focus on the activators and inhibitors of PKC and their applications in clinical trials.

## 2. Structure of PKC Isozymes

Several important review articles have already been reported regarding the structure of PKC isozymes [1,2,3]. All PKC isozymes consist of a regulatory domain containing the C1 and C2 domains, a catalytic (kinase) domain containing C3 (N-terminal lobe (N-lobe) domain) and C4 domain (C-terminal lobe (C-lobe) domain), and variable regions (V1–V5) (Figure 1). While the C1 domain of cPKCs and nPKCs interacts with diacylglycerol (DAG), the single C1 domain of aPKCs cannot bind to DAG. The C2 domain of cPKCs binds to Ca^2+^, but not the C2-like domain of nPKCs. The C3 domain contains an ATP-binding site, and the C4 domain has a substrate-binding site. Although all PKC isozymes do not contain the phosphatidylserine (PS)-binding domain, PS, either alone or with DAG and Ca^2+^, is essential for PKC activation [1,2,3].

The regulatory region of all PKC isozymes contains an autoinhibitory pseudosubstrate domain that inhibits kinase activity by interacting with the substrate binding site within the catalytic region. The catalytic domain contains three phosphorylation motifs: an activation loop, a turn motif, and a hydrophobic motif [1,2,3]. Moreover, aPKCs have a protein–protein-interacting region known as Phox and Bem 1 (PB1) at the N-terminus of the regulatory domain. The PB1 domain binds with partitioning-defective protein 6 (Par6), p62 (also known as sequestosome 1, SQSTM1), or mitogen-activated protein kinase kinase 5 (MEK5) [8,9].

## 3. PKC Inhibitors

Most of PKC inhibitors are C3 domain-binding inhibitors (ATP competitive PKC inhibitors), but C1 domain- (DAG competitive PKC inhibitors) and C4 domain-binding PKC inhibitors (substrate competitive PKC inhibitors) have also been reported. Among the C1-domain binding agents, bryostatin-1 acts as both an activator and inhibitor of PKC.

### 3.1. C1 Domain-Binding PKC Inhibitors (DAG Competitive PKC Inhibitors)

**Figure 2:**
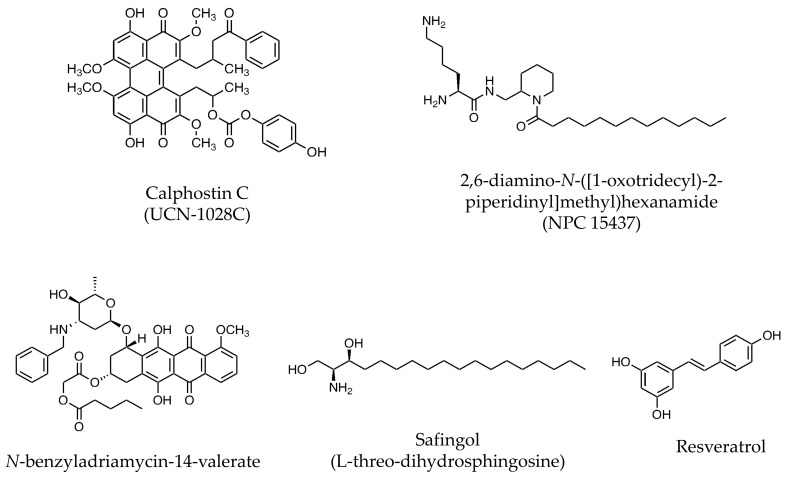
Chemical structure of C1 domain-binding PKC inhibitors (DAG competitive PKC inhibitors).

Several C1 domain-binding PKC inhibitors have been reported, such as calphostin C (UCN-1028C), a compound isolated from a dark pigmented mold, *Cladosporium cladosporioides* (half-maximum inhibitory concentration (IC_50_) = 0.05 μM for rat brain PKC) [10,11], 2,6-diamino-*N*-([1-oxotridecyl)-2-piperidinyl] methyl) hexanamide (NPC 15437), a synthetically derived compound (IC_50_ = 22 μM for PKCα [12] and 19 μM for rat brain PKC [13]), *N*-benzyladriamycin-14-valerate (AD 198; IC_50_ = 9 μM for the binding of phorbol 12, 13-dibutyrate (PDBu) to rat brain PKC), a lipophilic anthracycline [14], and resveratrol, a polyphenolic phytoaxelin present in dietary sources (IC_50_ = 2 μM for PKCα) [15] (Figure 2). There are no reports regarding the use of these inhibitors in clinical trials.

On the other hand, safingol (L-threo-dihydrosphingosine), a lyso-sphingolipid PKC inhibitor, can also interact with the C1 domain and inhibit the enzymatic activity and [^3^H] PDBu binding of purified rat brain PKC with similar IC_50_ values (37.5 and 31 μM, respectively) [16]. A pilot phase I study of safingol alone or in combination with doxorubicin in tumor-bearing animals suggested that safingol could be administered safely along with 45 mg/m^2^ of doxorubicin [17]. Furthermore, in a phase I clinical trial of safingol in combination with cisplatin in advanced solid tumors, the recommended phase II dose was safe (safingol (840 mg/m^2^) and cisplatin (60 mg/m^2^)) when administered every 3 weeks. However, in this phase I trial, safingol was used as an inhibitor of sphingosine kinase 1, which produces sphingosine 1-phosphate, that is associated with cancer cell growth and proliferation, and not as a PKC inhibitor [18]. Currently, a phase I clinical trial of safingol plus all-trans-*N*-(4-hydroxyphenyl) retinamide (fenretinide) is ongoing in patients with relapsed malignancies (NCT01553071).

### 3.2. C2 Domain-Binding PKC Inhibitors (Ca^2+^ Competitive PKC Inhibitors)

cPKC isozymes contain a C2 domain that binds to Ca^2+^, and the nPKC isozymes have a C2-like domain that cannot bind to Ca^2+^ but binds to phosphotyrosines [19]. There are no reports regarding PKC inhibitors that can block the interaction with the C2 domain. However, PKCβ C2 region-derived peptides, such as C2-1 (KQKTKTIK), C2-2 (MDPNGLSDPYVKL), and C2-4 (SLNPEWNET), inhibit the binding of PKCβ C2 fragment to the receptor for activated C-kinase (RACK). These peptide inhibitions specifically block the translocation and function of cPKC isozymes containing the C2 domain, but not nPKC isozymes containing the C2-like domain [20,21].

### 3.3. C3 Domain (N-Lobe Domain)-Binding PKC Inhibitors (ATP Competitive PKC Inhibitors)

Among PKC inhibitors, ATP competitive small molecule inhibitors have been broadly developed and applied in clinical trials. ATP competitive inhibitors interact with the ATP-binding pocket (C3 domain). Their IC_50_ values depend on the affinity of inhibitor and the amount of added ATP [22]. High sequence homology and structural similarity in the C3 domain of PKC isozymes are major obstacles in the development of PKC isozyme-specific inhibitors [3].

#### 3.3.1. Indolocarbazole Compounds

**Figure 3:**
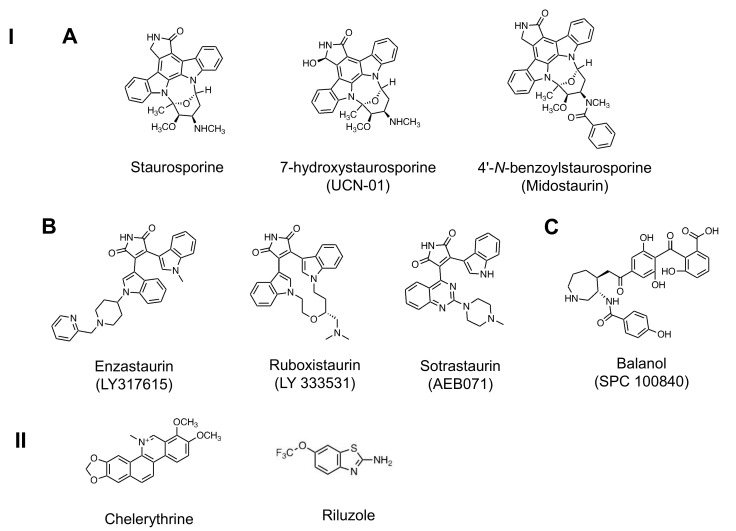
I. Chemical structure of C3 domain-binding inhibitors (ATP competitive PKC inhibitors): (**A**) indolocarbazole compounds, (**B**) Maleimide-based inhibitors. Bisindolylmaleimide (Bis) compounds (enzastaurin and ruboxistaurin) and sotrastaurin, and (**C**) other ATP competitive PKC inhibitor. II. Chemical structure of C4 domain-binding inhibitors (substrate competitive PKC inhibitors).

Several natural and biosynthetic indolocarbazole compounds have been identified, for example, staurosporine and its analogs such as Gö 6976, K252 compounds, 7-hydroxystaurosporine (UCN-01), and 4’-*N*-benzoylstaurosporine (midostaurin) (for review see [23,24,25,26]) (Figure 3). Among the staurosporine analogs, clinical trials of UCN-01 and midostaurin have been broadly carried out.

Staurosporine (C3): Staurosporine is a compound produced by *Streptomyces* sp. with an IC_50_ value of 2.7 nM for PKC and binds to its catalytic domain [27]. Its inhibitory activity increases in the following order: cPKC > nPKC > aPKC [28,29]. Staurosporine also shows inhibitory activity against several serine/threonine protein kinases and tyrosine kinases [25]. However, there are no reports on the use of staurosporine in clinical trials.

UCN-01 (7-hydroxystaurosporine) (C3): UCN-01 is a compound isolated from *Streptomyces* sp. (strain N-126), and its stereoisomer is UCN-02 (7-epi-hydroxystaurosporine) [30,31]. The IC_50_ values of UCN-01 and UCN-02 are 4.1 and 62 nM, respectively [31]. Similar to staurosporine, UCN-01 inhibits PKC activity by binding to its catalytic domain [32] and exhibits significantly higher affinity toward cPKC than nPKC and aPKC [28,29]. The elimination half-lives of UCN-01 after intravenous injection into mice, rats, and dogs were 3.00–3.98, 4.02–4.46, and 11.6 h, respectively [33].

CN-01 inhibits the growth of cancer cells by blocking the cell cycle progression from G1 to S phase that is important in regulating cell proliferation [34]. UCN-01-mediated inhibition of the G1 to S phase transition is caused due to the reduction of cyclin-dependent kinase 1/2 (CDK1/2) [35,36,37], cyclin A [37], and checkpoint kinase 1/2 (CHK1/2) [38,39] as well as the induction of CDK inhibitors p21 and p27 [36,37].

Phase I trials of UCN-01 have been performed, in combination with prednisone, in patients with solid cancers and lymphomas [40], with irinotecan in patients with solid cancers [41], with perifosine in patients with relapsed and refractory acute leukemias and high-risk myelodysplastic syndrome [42], with fludarabine monophosphate in patients with relapsed lymphoma [43], with cisplatin in advanced solid tumors [44], with fluorouracil in patients with advanced solid tumors [45], and with carboplatin in advanced solid tumors [46]. However, several studies have shown no objective response [40,41,42,45].

Furthermore, Phase I/II clinical trials of UCN-01 have been performed in combination with irinotecan in patients with metastatic triple negative breast cancer (TNBC: negative for estrogen receptor, progesterone receptor, and HER2) [47,48]. Although impressive clinical activity was not obtained, a phase II study reported that effective CHK1 inhibition could enhance chemotherapy-induced apoptosis in *TP53-*mutant tumors [48]. Phase I/II studies of UCN-01 and topotecan were performed in patients with advanced ovarian cancer [49,50], but significant clinical benefit was not observed in the phase II study [50]. No further clinical trials of UCN-01 have been conducted after these studies.

Midostaurin (4’-N-benzoylstaurosporine): Midostaurin (also known as PKC412; CGP 41251) is a staurosporine analog isolated from *Streptomyces staurosporeus*. Similar to staurosporine, midostaurin is an ATP-competitive inhibitor and inhibits multiple protein kinases. Although midostaurin has lower inhibitory activity for PKC, its specificity for PKC is higher compared with staurosporine [51,52]. Midostaurin treatment inhibits the growth of various cancer cells and reverses P-glycoprotein-mediated multidrug resistance of cancer cells by interfering with P-glycoprotein function [51,52].

FMS-like tyrosine kinase 3 (*FLT3*) mutations with internal tandem duplication (ITD) are associated with high leukemic burden and poor prognosis in patients with acute myeloid leukemia (AML) [53]. *FLT3*/ITD mutations stimulate the tyrosine kinase activity of FLT3, resulting in growth factor-independent proliferation of *FLT3*/ITD-mutant AML cells [53,54]. G1 arrest and apoptosis were observed in midostaurin-treated *FLT3*-mutant leukemia cells by direct inhibition of tyrosine kinase (IC_50_ ≤ 10 nM) [55]. In a recent phase III trial, the addition of midostaurin to standard chemotherapy significantly prolonged overall and event-free survival in mutant *FLT3*-positive AML patients [56].

Furthermore, systemic mastocytosis is a heterogeneous group of disorders caused by the abnormal accumulation of mast cells in organs, such as the bone marrow, liver, spleen, gastrointestinal tract, and skin. Most patients with systemic mastocytosis have an Asp816Val (D816V) mutation in the KIT receptor tyrosine kinase [57]. Midostaurin treatment significantly reduced the percentage of peripheral blood mast cells and serum histamine levels in patients with systemic mastocytosis through inhibition of KIT tyrosine kinase [58]. In addition, midostaurin induced apoptosis and downregulation of CD2 and CD63 [59] and inhibited IgE-dependent upregulation of CD63 in the mast cell leukemia cell line HMC-1 [60], resulting in enhanced inhibition of cell growth. A phase II trial showed significant clinical benefits in patients with advanced systemic mastocytosis after oral treatment with midostaurin [61,62]. However, no unexpected toxicity was observed with a median follow-up of 10 years after the phase II trial [61].

Midostaurin has been approved by the Food and Drug Administration (FDA) since April 2017 for the treatment of newly diagnosed adult AML patients with mutant *FLT3*-positive or adult patients with systemic mastocytosis with associated hematological neoplasm, or mast cell leukemia (https://www.fda.gov/drugs/resources-information-approved-drugs/midostaurin) (access on 10 September 2020). Midostaurin treatment indicated higher cost-effectiveness in mutant *FLT3*-positive adult AML patients compared to the standard of care in these patients [63]. In a phase II hypothesis-generating trial, the addition of midostaurin to intensive chemotherapy increased event-free survival at 2 years by 39% (95% confidence interval (CI), 33–47%) and 34% (95% CI, 24–47%) in younger and older patients, respectively, compared to historical controls treated within five prospective trials [64]. In addition, further clinical trials of midostaurin are in progress [65].

As a result, although midostaurin was originally developed as a PKC inhibitor, its success in clinical trials is mainly due to the inhibition of tyrosine kinase. However, it is not clear whether these midostaurin-induced positive results in AML patients are caused owing to inhibition of tyrosine kinase alone or both tyrosine kinase and PKC. Nevertheless, while it is true that midostaurin-mediated inhibition of tyrosine kinases is effective against AML, other tyrosine kinase inhibitors (e.g., gilteritinib and quizartinib) also show significantly improved clinical events in patients with *FLT3*-mutated AML [66,67].

#### 3.3.2. Maleimide-Based Inhibitors

##### Bisindolylmaleimide (Bis) Compounds

Bis compounds are also synthetic analogs of staurosporine. Examples of typical compounds include Bis-1 (also known as GF 109203X or Gö 6850) [68], Bis-8 (Ro 31-7549) [69], Bis-9 (Ro 31-8220) [69], enzastaurin (LY317615) [70], and ruboxistaurin (LY 333531) [71]. While Bis-1, -2, -3, -8, -9, and -10 exhibit high inhibitory potential for PKC, they can also suppress other protein kinases [68,69,72,73]. However, Bis-4 and -5 exhibit little or no inhibition of PKC as well as several other protein kinases [72]. On the other hand, enzastaurin and ruboxistaurin are selective inhibitors of PKCβ [70,71] and have been extensively applied in clinical trials.

Enzastaurin (LY317615): Enzastaurin is an acyclic bisindolylmaleimide and an ATP-competitive, selective inhibitor of PKCβ. The IC_50_ values were 6 nM for PKCβ, 39 nM for PKCα, 83 nM for PKCγ, and 110 nM for PKCε in a cell-free assay [70]. Several studies have reported that enzastaurin induces antiproliferative and proapoptotic activity by inhibiting AKT and GSK3β, a downstream target of the AKT pathway [70,74,75,76], in addition to inhibiting PKCβ [76,77].

Despite its inhibitory effects on cancer cells, a phase II trial of enzastaurin in combination with bevacizumab [78] and a phase III trial of enzastaurin alone [79] showed no clear clinical benefit in patients with recurrent malignant gliomas. In a phase III trial of enzastaurin, patients with high-risk diffuse large B-cell lymphoma (DLBCL) received a daily dose of enzastaurin (500 mg) orally for 3 years, but no significant improvement in disease-free survival was observed [80].

Furthermore, in a phase II trial of enzastaurin, there were no significant clinical benefits in patients, with previously treated multiple myeloma [81], with brain metastasis after whole brain radiotherapy [82], with epithelial ovarian or primary peritoneal carcinoma [83], with relapsed or refractory mantle cell lymphoma [84], with metastatic breast cancer previously treated with an anthracycline- and a taxane-containing regimen [85], and with relapsed or refractory advanced cutaneous T-cell lymphoma [86].

In addition, several phase II studies of enzastaurin in combination with other anticancer drugs have been conducted in patients with various cancers, such as erlotinib or erlotinib/ enzastaurin in patients with non-small-cell lung cancer (NSCLC) [87], temozolomide or temozolomide/enzastaurin plus radiation therapy in patients with glioblastoma multiforme and gliosarcoma [88], docetaxel/prednisone or docetaxel/prednisone/enzastaurin in patients with castration-resistant metastatic prostate cancer [89], paclitaxel/carboplatin or paclitaxel/carboplatin/enzastaurin in patients with advanced ovarian cancer [90], 5-fluorouracil/leucovorin plus bevacizumab with or without enzastaurin in patients with metastatic colorectal cancer [91], pemetrexed or pemetrexed/enzastaurin in patients with advanced NSCLC [92], and gemcitabine or gemcitabine/enzastaurin in patients with advanced or metastatic pancreatic cancer [93]. However, these phase II trials failed to show any clinical benefits (e.g., progression-free survival) in these combinatorial treatments.

A phase I trial has also been conducted in children with recurrent central nervous system malignancies [94]. Despite the absence of objective responses, enzastaurin was well tolerated in children and the recommended phase II dose is 440 mg/m^2^/day administered once, daily [94].

Ruboxistaurin (LY 333531): Ruboxistaurin, a macrocyclic bisindolylmaleimide compound, shows higher inhibitory activity for PKCβ1 (IC_50_ = 4.7 nM) and PKCβ2 (IC_50_ = 5.9 nM), compared to other PKC isozymes, through ATP-dependent competitive inhibition [71].

PKCβ is highly expressed in the retina. Ruboxistaurin reduces the pathogenesis of diabetic retinopathy in diabetic rats by inhibiting PKCβ and hence preventing the increase in leukostasis and decrease in retinal blood flow [95,96]. In addition, it reduced the expression of endothelin-1 and platelet-derived growth factor in the retina [97] and inhibited vascular endothelial growth factor-induced phosphorylation of Akt and extracellular signal-regulated kinase 1/2 [98].

Patients with diabetic nephropathy exhibit either a painless syndrome with loss of sensation or a painful disorder accompanied by hyperalgesia and allodynia [99,100]. Ruboxistaurin attenuates diabetic hyperalgesia in diabetic rats by reducing the neuronal nitric oxide synthase-cGMP system [101]. Ruboxistaurin also inhibits NADPH oxidase-mediated production of reactive oxygen species in the kidney of diabetic rats, which is associated with renal injury [102]. Ruboxistaurin (10 μM) binds to the ATP binding site of 3-phosphoinositide dependent protein kinase-1 (PDK1), which is involved in the insulin-like growth factor signaling pathway, and exhibits higher inhibitory effects on PDK1, compared to other bisindolylmaleimides (each 10 μM), such as Bis-1, -2, -3, and -8 [73].

Transforming growth factor-β (TGF-β) activation stimulates the phosphoinositide-3-kinase/Akt pathway that accelerates renal injury and dysfunction [103]. Ruboxistaurin treatment reduces high glucose-induced Akt and TGF-β activation in mesangial cells and Akt activation in the renal cortex of diabetic rats [104]. In addition, ruboxistaurin-treated rat models of diabetic nephropathy showed a significant decrease in osteopontin expression, in addition to macrophage infiltration, interstitial fibrosis, and TGF-β activity in tubular epithelial cells of the cortex [105]. Based on these results, ruboxistaurin has been considered as a potential therapeutic agent for diabetic nephropathy and retinopathy.

A phase III study investigated the effect of ruboxistaurin (32 mg/day) on vision loss in patients with moderate to severe non-proliferative diabetic retinopathy. Reduced occurrence of sustained moderate visual loss (≥15-letter decline in visual acuity sustained for the last 6 months of study participation) was observed in patients with greatest ruboxistaurin exposure (~5 years), compared to control patients (~2-year ruboxistaurin exposure) [106]. Furthermore, two phase III trials of ruboxistaurin have been conducted in patients with (Early Treatment Diabetic Retinopathy Study) retinopathy level 20 to 47D or 35B to 53E, and no prior panretinal or focal photocoagulation in at least one eye at baseline. Although ruboxistaurin treatment showed an approximately 50% reduction in sustained moderate vision loss, caused due to diabetic macular edema, statistical significance was not achieved [107]. For patients with diabetes and symptomatic diabetic peripheral neuropathy, two identical, phase III, parallel, randomized, double-blind, placebo-controlled trials of ruboxistaurin (32 mg/day) have been performed, but these trials failed to show a significant and progressive improvement in symptoms [108]. Based on these findings, ruboxistaurin has not been used for further clinical trials.

##### Sotrastaurin

Sotrastaurin {AEB071; 3-(1H-indol-3-yl)-4-[2-(4-methylpiperazin-1-yl) quinazolin-4-yl] pyrrole-2,5-dione} is a potent and selective pan-PKC inhibitor, with various K_i_ values for PKC isozymes, such as 0.95 nM for PKCα, 0.64 nM for PKCβ, 0.22 nM for PKCθ, and 1.8–3.2 mM for PKCδ, ε, and η [109,110]. Sotrastaurin exhibits immunosuppressive functions, such as inhibition of T-cell activation [111] and suppression of B-cell antibody response [112]. Sotrastaurin has been reported to prevent T-cell-mediated rejection in liver and kidney transplantation [113]. The efficacy and safety of sotrastaurin alone in de novo kidney transplant recipients [114], sotrastaurin plus tacrolimus in de novo liver [115], and kidney transplant recipients [116], and sotrastaurin plus everolimus in de novo kidney transplant recipients [117] were evaluated through phase II clinical trials. All these clinical trials exhibited adverse effects and high failure rates with respect to efficacy.

Furthermore, sotrastaurin showed growth inhibitory effects on CD79-mutant DLBCL through NF-κB pathway inhibition and induction of G_1_-phase cell-cycle arrest and/or cell death [118,119]. However, a phase Ib study of safety and efficacy of sotrastaurin and everolimus (mTOR inhibitor) in patients with CD79-mutant or activated B-cell-like subtype DLBCL exhibited suboptimal tolerability of the combination treatment, resulting in no implementation of phase II (NCT01854606).

In addition, a recent phase I study of sotrastaurin in patients with metastatic uveal melanoma showed that it was well tolerated, and modest clinical activity was observed, with a low objective response rate (3%) [120].

#### 3.3.3. Other ATP Competitive PKC Inhibitors

Balanol (SPC 100840) was isolated from *Verticillium balanoides* and shows IC_50_ values of 4–9 nM for PKCβ1, β2, γ, δ, ε, and η and 150 nM for PKCζ [121]. Several balanol analogs have also been synthesized [122,123,124]. For example, cyclopentane-based analogs of balanol are more potent PKC inhibitors than balanol alone [122,123].

Melittin (GIGAVLKVLTTGLPALISWIKRKRQQ) inhibits PKC activation with an IC_50_ of 3 μM [125] or 0.8 μM [126] through direct interaction with the MgATP-sensitive binding domain [125,127].

### 3.4. C4 Domain (C-Lobe Domain)-Binding PKC Inhibitors (Substrate Competitive PKC Inhibitors)

Pseudosubstrate-derived peptide inhibitors and mutant peptide inhibitors bind to the C4 domain of PKC. Some aPKC inhibitors (e.g., ICA-1 and ζ-Stat) also bind to the C4 domain.

#### 3.4.1. Peptide Inhibitors

PKC peptide inhibitors are mainly divided into (1) peptides derived from PKC protein fragment and (2) peptides obtained by the mutation of phosphorylation sites of PKC substrates. Myristoylated (myr-PKC) inhibitors show higher inhibitory effects on target PKC isozymes than non-myristoylated PKC peptide inhibitors [128,129,130,131]. Moreover, D-type amino acids are used to increase the inhibitory efficiency of peptide inhibitors [131,132]. Furthermore, peptide length can influence the potency of peptide inhibitor. Reduction in peptide length leads to decreased potency of the inhibitor [130,133].

PKC protein fragment-derived peptide inhibitors: As mentioned above, the regulatory region of PKCs contains an autoinhibitory pseudosubstrate domain that interacts with the C4 domain. These pseudosubstrate-derived peptides are used as selective and cell-permeable inhibitor of PKC, such as PKCα/β pseudosubstrate peptide (PKC19–36) (RFARKGALRQKNVHEVKN) and its derivative (FARKGALRQ) [128,133], PKCε pseudosubstrate peptide (ERMRPRKRQGAVRRRV) [134], and PKC*ζ* pseudosubstrate-derived *ζ*-inhibitory peptide (ZIP; SIYRRGARRWRKL) [135]. Although these PKC protein fragment-derived peptide inhibitors are a useful tool for understanding the PKC-mediated signaling pathway, they may not be suitable as therapeutic agents because of their weak inhibitory abilities for PKC [2].

Furthermore, several peptide inhibitors have been synthesized for inhibiting the translocation of the target PKC isozyme. These peptides are mainly derived from variable regions, such as the PKCε V1 region-derived peptide (EAVSLKPT) [136], PKCα V5 region-derived peptide (QLVIAN) [137], PKCβ1 V5 region-derived peptide (KLFIMN) [138], PKCβ2 V5 region-derived peptide (QEVIRN) [138], and PKCδ V1 region-derived peptide (SFNSYELGSL) [139]. As an exception, PKCβ C2 region-derived peptides can also inhibit the translocation of cPKC isozymes. PKCβ C2 region-derived peptides, such as C2-1 (KQKTKTIK), C2-2 (MDPNGLSDPYVKL), and C2-4 (SLNPEWNET), inhibit the binding of PKCβ C2 fragment to RACK. These inhibitions block RACK activation-induced translocation of PKCβ and thereby decrease PKCβ translocation-mediated function [20,21]. However, these peptides show poor cellular membrane penetration which becomes a potential obstacle to their clinical application. For their efficient cytosolic delivery, the grafting of cell-penetrating peptides (e.g., human immunodeficiency virus (HIV-1) Tat) has been broadly used [136,139,140,141].

In a phase II clinical trial of PKCδ V1 region-derived peptide (also known as delcasertib or KAI-9803) [139], its intravenous injection into patients within 6 h of undergoing primary percutaneous coronary intervention for acute ST elevation myocardial infarction did not improve clinical events and left ventricular function and did not reduce expression of biomarkers of myocardial injury [140]. Furthermore, in a phase II clinical trial of PKCε V1 region-derived peptides (KAI-1678) [136], its subcutaneous injection, for the treatment of neuropathic pain, in patients with postherpetic neuralgia failed to show a significant reduction in pain intensity ([141].

Mutant peptide inhibitors: Mutant peptide inhibitors are generated by replacing the phosphorylation sites (Ser or Thr) with mostly Ala [130,142]. However, a study has reported that Cys replacement instead of Ala increases the potency of the inhibitor [129]. Mutant peptide inhibitors block the binding of the substrate to PKC. However, these mutant peptide inhibitors show very low inhibitory efficiencies for PKC [130].

#### 3.4.2. Other Inhibitors Binding to the C4 Domain

Chelerythrine (IC_50_ = 0.66 μM), a natural benzophenanthridine alkaloid isolated from *Chelidonium majus*, is a competitive inhibitor with respect to the phosphate acceptor (histone IIIS) and a noncompetitive inhibitor with respect to ATP, meaning that it binds to the C4 domain of the PKC catalytic region [143]. It has broad biological activities, such as anticancer [144], anti-inflammatory [145], antiviral [146], antifungal [147], and antibacterial effects [148]. Chelerythrine inhibits the growth of cells in various ranges of IC_50_ values. For example, the IC_50_ of chelerythrine is 2.6–4.2 μM for TNBC cell lines (MDA-MB-231, BT-549, HCC1937 and MDA-MB-468) [149], >10 μM for non-TNBC cell lines (MCF7, ZR-75-1, SK-BR-3 and MDA-MB-453) [149], 6.2 μM for HeLa cells [150], and 5.0–7.8 μM for NSCLC cell lines (HCC827, SK-MES-1, and A459) [151]. On the other hand, a study suggested that, while chelerythrine could not inhibit PKC activity, it could stimulate PKC activity in the cytosolic fractions of rat and mouse brain tissues at concentrations of up to 100 μM [152].

Riluzole binds to the catalytic domain of PKC, but ATP concentrations do not affect riluzole-mediated PKC inhibition. This means that riluzole is not a competitive inhibitor of ATP and binds to the C4 domain [153]. Riluzole is an FDA-approved medication that has neuroprotective properties and is used to treat amyotrophic lateral sclerosis. PKC is activated in amyotrophic lateral sclerosis, and riluzole-mediated PKC inhibition may be involved in the neuroprotective mechanism [153,154]. Furthermore, riluzole (0.1–10 μM) inhibits VEGF-stimulated PKC βII activation and cell proliferation in bovine retinal endothelial cell and human umbilical vein endothelial cell cultures [155]. Riluzole (30 μM) also inhibits PKC activity in the membrane of cortical cells [153].

HIV-1 Tat-peptide (aa 48–60), with an IC_50_ value of 22 nM for PKCα, competes with the kinase substrates, but not with ATP [156]. Furthermore, the inhibitor 1H-imidazole-4-carboxamide, 5-amino-1-[2,3-dihydroxy-4-[(phosphonooxy) methyl] cyclopentyl-[1R-(1a,2b,3b,4a)], binds to the C4 domain of PKCλ/ι, but not other PKCs [157]. Bis-1 is an ATP competitive inhibitor [68], but also a substrate competitive inhibitor that interferes with the binding of the pseudosubstrate domain to the substrate recognition site [158].

## 4. Atypical PKC Inhibitors

There are few reports on PKC isozyme-specific inhibitors, but some aPKC-specific inhibitors have recently been reported and are summarized below. These inhibitors block the activity of aPKC by binding to either the PB domain that exists at the N-terminus of aPKC or to the catalytic domain (Figure 1). The therapeutic efficacy of aPKC inhibitors in patients is yet to be investigated in clinical trials.

### 4.1. ZIP (PB Domain)

While ZIP (SIYRRGARRWRKL) shows high binding affinity for aPKC, it can also bind to multiple PKC isozymes [135]. Its potential inhibitory activity for PKCι and PKCζ is nearly equal (*K_i_* (95% CI) = 1.43 and 1.7 μM, respectively) [159]. ZIP does not inhibit the catalytic activity of the kinase domain of PKCζ but prevents the interaction of the PB1 domain of PKCζ with that of p62 by binding to an acidic surface on the PB1 domain of p62 [160,161].

PKMζ, an N-terminal truncated isoform of PKC*ζ,* plays a critical role in the maintenance of long-term potentiation, long-term memory, and chronic pain [159,162,163,164]. ZIP is a candidate inhibitor for PKMζ. Despite its dependence on substrate and kinase concentrations, myr-ZIP completely inhibits PKMζ activity in the range of 5–10 μM and its IC_50_ value for PKMζ ranges from 0.076 to 2 μM [135,159,163,165]. On the other hand, ZIP-induced excitotoxic death of cultured neurons at 5–10 μM has been reported [166]. In animal disease models, ZIP administration alleviated or prevented pain-related disorders, such as chronic visceral pain [167] and neuropathic pain [168], and memory-related disorders, such as anxiety in autism [169] and fear-mediated anxiety [170,171]. However, there are no data on the application of ZIP inhibitor peptide in clinical trials.

### 4.2. Auranofin and Sodium Aurothiomalate (PB1 Domain)

**Figure 4:**
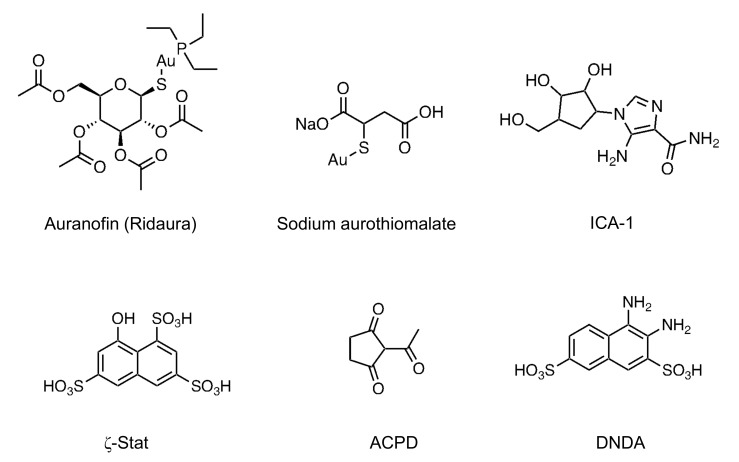
Chemical structure of atypical PKC inhibitors. These inhibitors show high potential inhibitory activity for PKCι/λ (PKCλ is the mouse homolog of PKCι) and PKCζ.

Gold compounds, auranofin (Ridaura; 1-thio-*β*-D-glucopyranosatotriethylphosphine gold-2,3,4,6-tetraacetate) and sodium aurothiomalate (Myochrysine; gold sodium thiomalate) (Figure 4) exhibit immunosuppressive activity and have been used in the treatment of rheumatoid arthritis [172,173]. They also inhibit PKCι and PKCζ signaling by selectively targeting the PB1 domain (Cys-69) of PKCι [174] and the PB1 domain (Cys-68) of PKCζ, respectively [175]. The binding of auranofin and sodium aurothiomalate to the PB1 domain blocks the interaction of PKCι and PKCζ with their adaptors, Par6, p62, and MEK5 [174,175]. For example, the sodium aurothiomalate inhibits the binding of PB1 domain of PKCζ and PKCι to Par6 with the IC_50_ values of 3 [175] and 1 µM [176], respectively. The cytotoxicity activity (IC_50_) of auranofin and sodium aurothiomalate is ≤10 μM in sensitive cancer cells and >40 μM in non-sensitive cancer cells [177,178,179]. Sodium aurothiomalate-sensitive cancer cells express significantly higher PKCι levels than insensitive cancer cells [179].

The phase I study of aurothiomalate has been conducted in patients with PKCι-overexpressed cancers, such as advanced NSCLC, ovarian cancer, and pancreatic cancer [180]. A feasibility study for enrolling asymptomatic ovarian cancer patients with increased levels of CA-125 (10 patients) has also been carried out by oral administration of auranofin, which resulted in decreased levels of CA-125 in one patient [181]. Furthermore, a phase I/II clinical trial of auranofin (NCT01419691) has been conducted in patients with chronic lymphocytic leukemia, small lymphocytic and prolymphocytic lymphoma [182].

### 4.3. ICA-1 (C4, C-Terminal Lobe Domain)

ICA-1 ([4-(5-amino-4-carbamoylimidazol-1-yl)-2,3-dihydroxycyclopentyl] methyl dihydrogen phosphate) specifically inhibits PKCι but not PKCζ [183]. Its binding pocket was identified in the C-lobe of the catalytic (kinase) domain [183,184]. ICA-1 inhibited the activity of PKCι for myelin basic protein (MBP) by 16% at 0.1 μM, 25% at 1 μM, and 45% at 5 μM, and for phosphotransferase by 22.6% at 0.1 μM, 53.5% at 1 μM, and 80.0% at 5 μM. However, it showed no effects on the activity of PKCζ for MBP and phosphotransferase [183]. In an inhibitory study using the human neuroblastoma cell line BE(2)-C, ICA-1 and aurothiomalate reduced cell proliferation by 58% at 48 h with an IC_50_ value of 0.1 and 100 μM, respectively [183]. Although ICA-1-mediated reduction in cell proliferation in several cancer cells [183,184,185] or in cancer animal models [186] has been reported, there are no studies on its application in the clinical trials.

### 4.4. ζ-Stat (C4, C-Lobe)

ζ-Stat (8-hydroxy-1,3,6-naphthalenetrisulfonic acid) is regarded as a PKCζ specific inhibitor. Similar to ICA-1, its biding site exists in the C-lobe of the kinase domain [184]. When MBP was incubated with recombinant active PKCι and PKCζ in the presence of ζ-Stat (1–20 μM), ζ-Stat showed 51% inhibition of PKCζ at 5 μM, but only 13% inhibition of PKCι at 20 μM [184]. Recently, ζ-Stat was used to elucidate the signaling mechanisms of PKCζ-mediated growth, proliferation, and metastasis of cancer cells [184,187,188].

### 4.5. ACPD and DNDA (C4, C-Lobe; ACPD C3, N-Lobe for PKC-ζ)

ACPD (2-acetyl-1,3-cyclopentanedione) and DNDA (3,4-diaminonaphthalene-2,7-disulfonic acid) bind to the catalytic domain of both PKCι and PKCζ and inhibit both these kinases [189]. In more detail, ACPD interacts with amino acid residues 469–488 of the catalytic domain of PKC-ι and amino acid residues 265–290 of PKC-ζ. DNDA interacts with amino acid residues 339–395 of PKC-ι and amino acid residues 337–393 of PKC-ζ [189]. The IC_50_ value of ACPD and DNDA were approximately 2.5 μM, which was calculated based on their inhibitory effects on cell proliferation [187]. ACPD and DNDA can be used to inhibit aPKC-mediated cancer cell proliferation and for understanding the aPKC-mediated signaling transduction pathway [185,189,190,191].

## 5. Antisense Oligonucleotides

PKCα is considered as a target for cancer treatment since it is overexpressed in many cancer cells [5,192]. A PKCα-specific antisense oligonucleotide, aprinocarsen (ISIS3521/LY900003; a 20-base antisense oligonucleotide), has been applied in Phase II or/and III clinical trials in patients with recurrent high-grade astrocytomas [193], advanced NSCLC [194,195], advanced ovarian carcinoma [196], hormone-refractory prostate cancer [197], metastatic colorectal cancer [198], and previously treated low-grade non-Hodgkin’s lymphoma (NHL) [199]. Unfortunately, however, aprinocarsen treatment with or without anticancer drugs failed to exhibit significant survival and other clinical benefit [193,194,195,196,197,198,199].

## 6. PKC Activators

**Figure 5:**
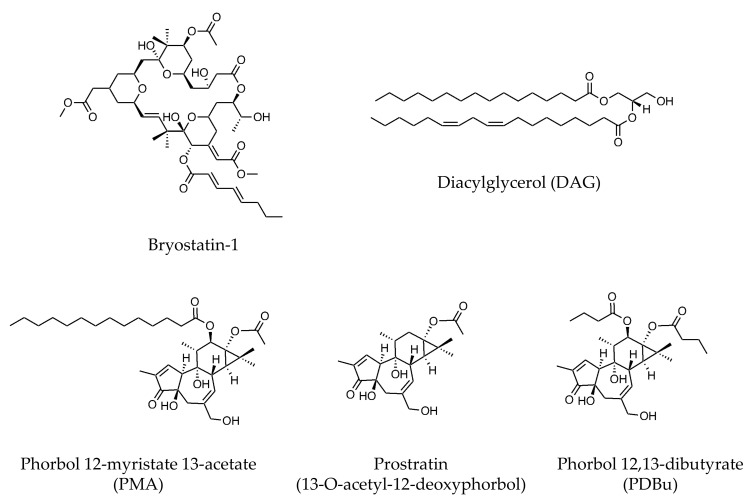
Chemical structure of PKC activators (C1 domain-binding ligands).

C1 domain ligands are universally used for PKC activation, including bryostatin-1 and DAG and its analogues such as PDBu, phorbol 12-myristate 13-acetate (PMA; also known as 12-O-tetradecanoylphorbol-13-acetate (TPA)), and prostratin (13-O-acetyl-12-deoxyphorbol) (Figure 5). Despite two phase I trials of PMA to identify its dose tolerance [200,201], bryostatin-1 is the most widely used agent in clinical trials.

### 6.1. Bryostatin-1

Bryostatin-1, a macrocyclic lactone isolated from a marine invertebrate, binds to the C1 domain of PKC and acts both as an activator and inhibitor for PKC. For example, short-term exposure to bryostatin-1 stimulates PKC activation, while long-term exposure promotes downregulation of PKC activity [202].

#### 6.1.1. Bryostatin-1 as a PKC Inhibitor

Bryostatin-1 competes with cancer-promoting PKC ligands (e.g., DAG and phorbol esters) to bind to PKC since it has the same binding site (C1 domain) as PKC ligands. Based on these functions, phase II studies of bryostatin-1 with other anticancer drugs have been performed in patients with various cancers. Phase II trials of single-agent bryostatin-1 showed no clinical effects in several cancers, such as metastatic malignant melanoma [203,204,205], metastatic renal cell carcinoma [206,207], metastatic colorectal cancer [208], NHL [209], relapsed multiple myeloma [210], advanced sarcoma and advanced head and neck cancer [211], metastatic or recurrent squamous cell carcinoma of the head and neck [212], squamous cell carcinoma of the cervix [213], and recurrent epithelial ovarian carcinoma [214].

Furthermore, no clinical responses were observed in phase II studies of bryostatin-1/paclitaxel in patients with advanced pancreatic carcinoma [215], advanced NSCLC [216], and advanced or recurrent carcinoma of the cervix [217], as well as in a phase II study of four different doses of bryostatin-1/interleukin-2 treatment in patients with renal cell carcinoma [218].

On the other hand, bryostatin-1/paclitaxel treatment in a phase II study resulted in a superior response rate in patients with untreated, advanced gastric or gastroesophageal junction adenocarcinoma, compared to paclitaxel alone [219]. Another phase II study of the same treatment in patients with advanced esophageal cancer, despite potential anti-tumor activity, was prematurely closed because of excessive toxicity [220].

In patients with recurrent platinum-sensitive or resistant ovarian cancer, a phase II trial of bryostatin-1/cisplatin showed a modest response rate. However, it increased toxicity in platinum-pretreated patients [221]. A phase II study of bryostatin-1/vincristine showed efficacy in select patients (overall response rate of 31%) with aggressive B-cell NHL which relapsed after autologous stem cell transplantation [222].

From the clinical studies mentioned above, the doses used in clinical trials of single-agent bryostatin-1 ranged from 25 to 120 μg/m^2^ and from 25 to 65 μg/m^2^ in combination with other anticancer drugs. Interestingly, excessive toxicity was observed in patients treated with paclitaxel (80–90 μg/m^2^) plus bryostatin-1 (40–50 μg/m^2^) [220]. Furthermore, the combination of 45 μg/m^2^ bryostatin-1 and 50 μg/m^2^ cisplatin showed increased toxicity in platinum-pretreated patients [221].

#### 6.1.2. Bryostatin-1 as a PKC Activator

Previous studies have suggested that bryostatin-1- [223,224] or PMA- [225] mediated PKCε activation could reduce amyloid-β levels and prevent learning and memory deficits in mice with AD. In a single-dose (25 μg/m^2^) phase IIa clinical trial, bryostatin-1 administration to patients with AD showed cognitive improvement in the first 24 weeks through elevated PKCε levels [226]. A recent phase II study of bryostatin-1 (20 μg) in patients with AD suggested that the primary endpoint at 13 weeks showed no significance for the full analysis set (FAS), but the improved signals of Severe Impairment Battery (SIB) scores were obtained at 13 weeks for the Completers Set and for both data sets (FAS + SIB) at 15 weeks, compared to those of placebo patients [227].

Furthermore, several studies showed that bryostatin-1-mediated PKC activation could reactivate latent (inactive) HIV-1 [228,229]. However, a phase I clinical trial of bryostatin-1 (20 μg/m^2^) in HIV-1infected patients exhibited no effect on PKC activity or on the transcription of latent HIV-1. These negative results may be due to low plasma concentrations of bryostatin-1 [230].

## 7. Perspectives for Research and Application of PKC Inhibitors and Activators

PKCs are regarded as attractive targets for cancer therapy because their hyperactivation in many cancers [76,141,179,196]. For these reasons, clinical trials of PKC inhibitors have focused on the treatment of many cancers. However, PKCs are also associated with various diseases, such as neurological diseases, cardiovascular diseases, and infection. Activators and inhibitors of PKC can be used for the treatment of these diseases.

PKC and AD: As briefly mentioned above, recently, PKC activation has been attracting attention as a novel therapeutic strategy for AD. For example, reduced PKCε levels, but increased β-amyloid (Aβ) levels, were found in the hippocampus and temporal pole areas of patients with AD [231]. PKCε promotes the expression of brain-derived neurotrophic factor (BDNF) in the brain, which plays a role in the growth and maintenance of neuronal networks. However, reduced expression of PKCε and BDNF has been observed in the hippocampal neuron in patients with AD [224]. In fact, a phase IIa clinical trial showed that bryostatin-1-mediated activation of PKCε could result in cognitive improvement in the first 24 weeks [226]. In addition, PKCα activation can be a useful tool for treating AD [232].

In contrast, aPKC activation promotes the phosphorylation of β-site amyloid precursor protein (APP)-cleaving enzyme 1 (BACE1) at Ser498, which increases the Aβ generation during AD pathogenesis [233]. Inhibition of aPKCs reduced the levels of Aβ_1–40/42_ and phospho-tau in the brain of Het-MλKO mice treated with insulin [234]. Furthermore, PKCδ inhibition reduces BACE1 expression, Aβ levels, and neuritic plaque formation, and rescues cognitive deficits in an APP Swedish mutations K594N/M595L/presenilin-1 with an exon 9 deletion–transgenic AD mouse model [235].

These results suggest that the biological function of PKC isozyme in AD progression could be different, and that PKC might be a therapeutic potential target for AD.

PKC and HIV: Recently, PKC activators have received attention as latency-reversing agents in HIV treatment. They reactivate latent HIV-1 within immune cells (e.g., CD4 and CD8 T cells) through activation of the NF-κB transcription factor pathway and enhance the recognition and removal of HIV by the immune system [236,237]. Despite the failure of a phase I clinical trial using the PKC activator bryostatin-1, which could be due to its low plasma concentrations [230], PKC activators are still regarded as one of the most promising agents for reversing HIV-1 latency. Therefore, PKC activators may remarkably increase therapeutic efficacy of HIV in combination with antiretroviral drugs [238,239].

PKC, cardiac disease, and heart failure: PKCs are good therapeutic targets for the treatment of cardiac disease and heart failure. For example, PKCα, PKCβ, PKCδ, and PKCε are targeted for treating cardiac hypertrophy, PKCβ, PKCδ, and PKCε are targeted for treating heart failure, and PKCθ for lowing heart transplant rejection [6,240,241]. However, there are no reports regarding clinical trials of PKC activators or inhibitors for cardiac disease and heart failure, except phase I/II trials of delcasertib in patients with myocardial infarction [140,242].

## 8. Summary and Overall Conclusions

Activators and inhibitors of PKC and their applications in clinical trials are summarized in Table 1. Despite many clinical trials of PKC inhibitors in cancers, most of them showed no significant clinical benefits. On the other hand, a phase III trial of midostaurin plus standard chemotherapy in mutant *FLT3*-positive AML patients [56] and a phase II trial of midostaurin alone in patients with advanced systemic mastocytosis [61] exhibited significant clinical benefits, such as enhanced overall response rate, prolonged event-free survival, and low unexpected toxicity. These clinical benefits of midostaurin are mainly due to the inhibition of tyrosine kinase, but not PKC, as mentioned above. Further studies are needed to investigate whether these midostaurin-induced clinical benefits are caused by inhibition of tyrosine kinase alone or both tyrosine kinase and PKC. However, PKC inhibitors may increase clinical efficacy in combination with tyrosine kinase inhibitors.

The reason why PKC-targeted inhibitors show no significant clinical benefits in several clinical trials of cancers is not clear yet. However, we speculate that the following three possibilities might contribute to little or no clinical benefits in clinical trials of PKC inhibitors:

(1) Isozyme-nonspecific PKC inhibitors. PKC isozymes are involved in multiple biological functions in cancer cells, such as tumorigenic or anti-tumorigenic, pro-apoptotic or anti-apoptotic, and pro-proliferative or anti-proliferative [243,244]. Isozyme-nonspecific PKC inhibitors, especially, ATP competitive PKC inhibitors, can block the activation of PKC isozymes with both tumorigenic, anti-apoptotic, and pro-proliferative function as well as anti-tumorigenic, pro-apoptotic, and anti-proliferative function. Therefore, the use of isozyme-nonspecific PKC inhibitors as chemotherapy drugs could lead to decreased therapeutic efficacy in cancers.

(2) PKC mutations. PKC loss-of-function mutations are found in a multitude of cancers [2,244]. This suggests that PKC inhibitors fail to exhibit significant clinical benefits in patients with PKC-mutated cancers. However, there are no reports that show whether PKC mutations in cancers have been investigated in clinical trials of PKC inhibitors.

(3) Limitation of PKC as therapeutic target. PKCs related to pro-proliferative and anti-apoptotic function are significantly activated in cancers. Although the activation of these PKCs is inhibited by PKC inhibitors, other cellular signals (e.g., AKT) that are pro-proliferative and anti-apoptotic in cancer may be substituted for PKCs. On the other hand, certain inhibitors of PI3Ks that are upstream of PKCs and AKT show significant clinical benefits in cancer treatment [245,246,247].

Therefore, so long as cellular signals that have similar functions as PKCs are activated in cancers, the inhibition of PKC alone may result in little or no clinical benefits in clinical trials. In fact, accumulating evidence suggests that there is a limitation to design a cancer therapeutic strategy targeting PKC alone (Table 1).

## Figures and Tables

**Table 1:** Activators and inhibitors of protein kinase C (PKC) and their applications in clinical trials.

Diseases	PKC Activators or Inhibitors/Other Agents	Phases	Clinical Benefits	References
Cancer				
Metastatic triple negative breast cancer	UCN-01/irinotecan	II	No significant clinical benefits	[48]
Advanced ovarian cancer	UCN-01/topotecan	II	No significant clinical benefits	[51]
Mutant *FLT3*-positive acute myeloid leukemia	Midostaurin/standard chemotherapy	III	Significantly prolonged overall and event-free survival	[56]
Advanced systemic mastocytosis	Midostaurin	II	Significant clinical benefits and no unexpected toxicity	[61]
High-risk diffuse large B-cell lymphoma	Enzastaurin (LY317615)	III	No significant clinical benefits	[80]
Multiple myeloma, lung cancer with brain metastases, epithelial ovarian or primary peritoneal carcinoma, metastatic breast cancer, and relapsed or refractory mantle cell lymphoma and advanced cutaneous T-cell lymphoma	Enzastaurin	II	No significant clinical benefits	[81,82,83,84,85,86]
Advanced non-small-cell lung cancer	Enzastaurin/erlotinib	II	No significant clinical benefits	[87]
Glioblastoma multiforme and gliosarcoma	Enzastaurin/temozolomide + radiation therapy	II	No significant clinical benefits	[88]
Castration-resistant metastatic prostate cancer	Enzastaurin/docetaxel + prednisone	II	No significant clinical benefits	[89]
Advanced ovarian cancer	Enzastaurin/paclitaxel + carboplatin	II	No significant clinical benefits	[90]
Metastatic colorectal cancer	Enzastaurin/5-fluorouracil + leucovorin + bevacizumab	II	No significant clinical benefits	[91]
Advanced non-small cell lung cancer	Enzastaurin/pemetrexed	II	No significant clinical benefits	[92]
Advanced or metastatic pancreatic cancer	Enzastaurin/gemcitabine	II	No significant clinical benefits	[93]
Metastatic malignant melanoma, renal cell carcinoma, and colorectal cancer, non-Hodgkin’s lymphoma, relapsed multiple myeloma, advanced sarcoma and advanced head and neck cancer, metastatic or recurrent squamous cell carcinoma of the head and neck, squamous cell carcinoma of the cervix, and recurrent epithelial ovarian carcinoma	Bryostatin-1	II	No significant clinical benefits	[205,206,207,208,209,210,211,212,213,214]
Advanced pancreatic carcinoma, non-small cell lung cancer, and esophageal cancer, advanced or recurrent carcinoma of the cervix, and advanced gastric or gastroesophageal junction adenocarcinoma	Bryostatin-1/paclitaxel	II	No significant clinical benefits	[215,216,217,219,220]
Renal cell carcinoma	Bryostatin-1/interleukin-2	II	No significant clinical benefits	[218]
Recurrent platinum-sensitive or resistant ovarian cancer	Bryostatin-1/cisplatin	II	Modest response rate but high toxicity in platinum-pretreated patients	[221]
Aggressive B-cell non-Hodgkin lymphoma relapsing after autologous stem cell transplantation	Bryostatin-1/vincristine	II	Overall response rate of 31% (efficacy in select patients)	[222]
Diabetic retinopathy and neuropathy				
Moderate to severe non-proliferative diabetic retinopathy	Ruboxistaurin (LY 333531)	III	Reduced occurrence of sustained moderate vision loss but not significant	[106]
Diabetic retinopathy (retinopathy level 20 to 47D or 35B to 53E)	Ruboxistaurin	III	Approximately 50% reduction of sustained moderate vision loss but not significant	[107]
Diabetes and symptomatic diabetic peripheral neuropathy	Ruboxistaurin	III	No significant and progressive improvement in symptoms	[108]
Neurological diseases				
Alzheimer’s disease	Bryostatin-1	IIa	Cognitive improvement in the first 24 weeks	[226]
	Bryostatin-1	II	Improved the full analysis set and the Severe Impairment Battery scores at 15 weeks	[227]
Postherpetic neuralgia	KAI-1678	II	No significant reduction in pain intensity	[141]
Transplant rejection				
De novo kidney transplantation	Sotrastaurin (AEB071)	II	Low efficacy and high adverse events	[114]
De novo kidney transplantation	Sotrastaurin/tacrolimus	II	Limited benefits over standard immunosuppressive therapy and high adverse events	[116]
De novo kidney transplantation	Sotrastaurin/everolimus	II	High efficacy failure rates and adverse events	[117]
De novo liver transplantation	Sotrastaurin/tacrolimus	II	High efficacy failure rates and adverse events	[115]
Cardiovascular diseases				
Myocardial infarction	Delcasertib (KAI-9803)	II	No significant clinical benefits	[140]
Infections				
Human immunodeficiency virus (HIV) infection	Bryostatin-1	I	No effect on the transcription of latent HIV-1	[230]

## Data Availability

Not applicable.
